# A Software Tool for High-Throughput Real-Time Measurement of Intensity-Based Ratio-Metric FRET

**DOI:** 10.3390/cells8121541

**Published:** 2019-11-29

**Authors:** Masoud Ramuz, Alveera Hasan, Lena Gruscheski, Ivan Diakonov, Nikoleta Pavlaki, Viacheslav O. Nikolaev, Sian Harding, Chris Dunsby, Julia Gorelik

**Affiliations:** 1 Imperial College London, National Heart and Lung Institute, Du Cane Road, London W12 0NN, UK; m.ramuz17@imperial.ac.uk (M.R.); alveera.hasan13@imperial.ac.uk (A.H.); l.gruscheski@imperial.ac.uk (L.G.); i.diakonov@imperial.ac.uk (I.D.); sian.harding@imperial.ac.uk (S.H.); 2 Institute for Experimental Cardiovascular Research, University Medical Center Hamburg-Eppendorf, Martinistr. 52, D-20246 Hamburg, Germany; n.pavlaki@uke.de (N.P.); v.nikolaev@uke.de (V.O.N.); 3 Photonics Group, Department of Physics, and Centre for Pathology, Department of Medicine, Imperial College London, South Kensington Campus, London SW7 2AZ, UK; christopher.dunsby@imperial.ac.uk

**Keywords:** FRET, cAMP, signalling, microscopy

## Abstract

Förster resonance energy transfer (FRET) is increasingly used for non-invasive measurement of fluorescently tagged molecules in live cells. In this study, we have developed a freely available software tool MultiFRET, which, together with the use of a motorised microscope stage, allows multiple single cells to be studied in one experiment. MultiFRET is a Java plugin for Micro-Manager software, which provides real-time calculations of ratio-metric signals during acquisition and can simultaneously record from multiple cells in the same experiment. It can also make other custom-determined live calculations that can be easily exported to Excel at the end of the experiment. It is flexible and can work with multiple spectral acquisition channels. We validated this software by comparing the output of MultiFRET to that of a previously established and well-documented method for live ratio-metric FRET experiments and found no significant difference between the data produced with the use of the new MultiFRET and other methods. In this validation, we used several cAMP FRET sensors and cell models: i) isolated adult cardiomyocytes from transgenic mice expressing the cytosolic epac1-camps and targeted pmEpac1 and Epac1-PLN sensors, ii) isolated neonatal mouse cardiomyocytes transfected with the AKAP79-CUTie sensor, and iii) human induced pluripotent stem cell-derived cardiomyocytes (hiPSC-CM) transfected with the Epac-S^H74^ sensor. The MultiFRET plugin is an open source freely available package that can be used in a wide area of live cell imaging when live ratio-metric calculations are required.

## 1. Introduction

One of the major experimental methods in cellular biology for investigating the interactions, dynamics and localisation of specific molecules is through the measurement of luminescent or fluorescent probes [1]. The luminescent/fluorescent molecule either is attached to a molecule of interest, allowing characterisation of its location and function, or can be used in a sensor molecule that reports a specific local environmental parameter. An example of the latter technique is intermolecular Förster Resonance Energy Transfer (FRET). FRET works by the electronic excitation of a first fluorophore, the so-called donor fluorophore. The donor may then transfer its energy non-radiatively to a second acceptor fluorophore if they are separated by less than ~10 nm. The efficiency of energy transfer is inversely proportional to the sixth power of the distance between the two fluorophores [2,3]. In addition, FRET requires sufficient overlap of excitation spectrum of the acceptor with the emission spectrum of the donor and also depends on the relative angular orientations of the donor and acceptor dipoles [4].

In the case of heteroFRET, the two fluorophores are not identical and emit at different wavelengths [5]. There are a very large number of approaches to analysing spectral ratio-metric FRET data [6,7], the simplest of which is a straight-forward ratio of sensitized acceptor emission to the directly excited emission from the donor fluorophore. This simple calculation of the FRET ratio is useful for real-time monitoring of biosensor readouts in live cells and tissue slices.

Over recent years, a number of non-proprietary software packages have been written for ratio-metric FRET analysis, however their use is limited to analysis of previously saved image-stacks [8,9,10,11]. Some proprietary software offers real-time measurements of the ratios between fluorescent intensities in two channels; one example is MetaFluor (Molecular Devices, San Jose, USA) [12]. This provides additional flexibility, allowing experimental procedures to be carried out in response to changes in the FRET ratio. As a low-cost alternative, Julia U. Sprenger et al. (2012) built a customised epifluorescence FRET imaging system and developed a non-proprietary ImageJ macro, which displays real-time ratio-metric data obtained from a Micro-Manager controlled time-lapse acquisition of images of two fluorescence channels [13]. This macro gives a rough estimation of FRET ratios in real-time during the experiment, but it does not save the ratio data, and the method still requires a separate analysis of the image-stacks once they are saved.

Here we report a microscope system incorporating a motorized sample stage and describe a new software plug-in for Micro-Manager named MultiFRET, which allows for higher throughput real-time ratio-metric experiments. We experimentally demonstrated the capabilities of MultiFRET by measuring FRET responses in three different cell models that were stimulated to produce cAMP: i) isolated adult cardiomyocytes from transgenic mice expressing the cytosolic epac1-camps sensor, as well as its targeted version pmEpac1 and Epac1-PLN, ii) isolated neonatal mouse cardiomyocytes transfected with the AKAP79-CUTie sensor, and iii) human induced pluripotent stem cell derived cardiomyocytes (hiPSC-CM) transfected with the Epac-S^H74^ sensor. We used the conventional stimulants isoproterenol and 3-isobutyl-1-methylxanthine (IBMX) to increase levels of cAMP, a signalling molecule that is vital for regulation of cardiomyocyte contraction [14]. We compared our data to data obtained using the lower-throughput set of macros described in the J. U. Sprenger (2012) paper [13].

## 2. Materials and Methods

### 2.1. Adult Mouse Cardiomyocyte Isolation

All procedures were carried out under the EU2012 regulations. The protocol as described by P.T. Wright (2014) was followed to isolate cardiomyocytes [15]. Adult mice were sacrificed through cervical dislocation after brief isoflurane anaesthesia (Approval number from Hamburg is ORG741). The heart was removed and placed into cold HEPES buffer (NaCl 113 mM, KCl 4.7 mM, KH_2_PO_4_ 0.6 mM, Na_2_HPO_4_ 0.6 mM, MgSO_4_ 1.2 mM, NaHCO_3_ 12 mM, KHCO_3_ 10 mM, HEPES 10 mM and taurine 30 mM). The aorta was cannulated, and the heart perfused with HEPES buffer at 37 °C. Trypsin/liberase solution (HEPES plus 13 μM CaCl_2_, liberase 0.05 mg/mL, 0.3 mg/mL trypsin) was added to the perfusate after exiting fluid became clear. After 10 min, the heart was diced, shaken and triturated with a needleless 1mL syringe until all material had broken down. The suspension was centrifuged, the pellet was resuspended in blocking buffer (HEPES buffer plus 1%BSA and 50 μM CaCl_2_), and the cells were then washed and maintained in myocyte culture medium (MEM no L-glutamine, BSA 0.1%, penicillin/streptomycin 1%, L-glutamine 2 mM, BDM 10 mM and ITS-Supplement 1x).

### 2.2. Neonatal Mouse Cardiomyocyte Isolation

Neonatal ventricular mouse cardiomyocytes were isolated from one to two-day old pups according to the protocol provided by the manufacturer (Miltenyi Biotech, Bergisch Gladbach, Germany) (www.miltenyibiotec.com/protocols). The pups were sacrificed by cervical dislocation followed by decapitation. The hearts were excised using sterilised scissors and forceps, and were washed in Hank’s buffer salt solution (HBSS) to remove blood. The ventricles were minced into 1mm cubes and harvested tissue was transferred into a gentleMACS C tube containing 2.5 mL enzyme mix before being incubated at 37 °C for 15 min. The tissue fragments were subjected to a mechanical dissociation process using a gentleMACS dissociator (Miltenyi Biotech, Bergisch Gladbach, Germany). This step (enzymatic digestion and tissue dissociation) was repeated three times. After the third time, 7.5 mL of culture medium 199 (M199), supplemented with 10% (v/v) FCS, 1% (v/v) VitB12, 1% (v/v) L- glutamine, 200 µg/mL streptomycin and 200 U/mL penicillin, was added into the tube before passing the sample through a 70 µm gauge mesh filter insert to remove large particles. The filtrate was centrifuged at 1000 rpm for 5 min, and the supernatant was removed. The pellet was suspended in fresh supplemented M199 and incubated for one hour at 37 °C in 1% CO_2_ to allow for a separation between the cardiac fibroblasts and cardiomyocytes (fibroblasts adhere faster to the surface of the flask compared to the cardiomyocytes). After one hour, the medium with cardiomyocytes was collected in a tube and centrifuged at 1000 rpm before being counted and plated onto coverslips.

### 2.3. Generating HiPSC-Derived Cardiomyocytes

The human IMR-90 stem cell line (Wicell, Madison, WI, USA) was differentiated using the Burridge and Denning protocol to generate cardiomyocytes. HiPSC’s were plated onto Matrigel-coated plastic culture dishes and maintained in E8 media (Stem Cell Technologies Inc. Cambidge, UK) until they were ready for differentiation (D0) [16]. D0–1: 6 µM CHIR99021 (Bio-Techne Ltd., Abingdon, UK), D2: RPMI medium (Sigma-Aldrich Company Ltd, Gillingham, Dorset, UK) with insulin-negative B27 supplement (RB-) (Fisher Scientific, Loughborough, UK), D3–4: C59 2.5 µM (Bio-Techne Ltd., Abingdon, UK), D5–10: RB-, D11–14: metabolic selection with RPMI-1640 without glucose, D15: RPMI with insulin positive B27 supplement (RB+). On D15, the CM were dissociated and re-plated to glass-bottomed MatTek dishes and cultured in RB+ until Day 30 for experimental use.

### 2.4. Sensors Used

For the validation experiments, five different sensors were used (Table 1). The epac1-camps sensor contains a truncated EPAC1 protein with only the cAMP binding domain, flanked by the ECFP (ex. 434 nm, em. 477 nm) and Venus (ex. 515 nm, em. 528 nm) fluorophores (see Table 1). The cAMP sensor Epac1-camps is well-documented within our lab and shows a high sensitivity to isoproterenol, PDE inhibitors and AC stimulation. We additionally used the Epac-S^H74^ sensor, which contains a mutated full-length EPAC1 protein as a sensor, flanked by mTurquoise (ex. 434 nm, em. 474 nm) as a donor fluorophore and a Venus dimer as an acceptor. Furthermore, we used two localised versions of the epac1-camps sensor expressed in transgenic mice. One of these is named pmEpac1 and is targeted to the plasma membrane, while the other is named Epac1-PLN and is localised at the sarcoplasmic reticulum. We also used a localised AKAP79-targetted CUTie sensor (kind gift from Manuela Zaccolo, Oxford university, Oxford, UK), which has the cAMP-binding domain of the protein kinase A regulatory subunit type II beta and uses ECFP as the donor and EYFP (ex. 513 nm, em. 527 nm) as the acceptor. Importantly, upon binding of cAMP, the AKAP79-CUTie sensor shows an increase in FRET, while the other two sensors show a decrease in FRET.

### 2.5. Transfection

HiPSC-CM were transfected with the cAMP-specific Epac-S^H74^ sensor FRET sensor [20] using Lipofectamine3000 (Fisher Scientific, Loughborough, UK) in Opti-MEM (thermo Fisher) as per the manufacturer’s guidelines and incubated for 2 days before use. In the same way, neonatal mouse CMs were transfected with the AKAP79-CUTie sensor.

### 2.6. Microscopy System

Our microscopy system is based on the same principles as shown by J. U. Sprenger et al. [13], but with the addition of a mechanised sample stage and focus drive (Märzhäuser Wetzlar, Wetzlar, Germany) that allow higher throughput acquisition of multiple fields of view (FOV) in one experiment. Individual cell positions (X, Y and Z) are recorded within the Micro-Manager prior to an experiment, allowing each image capture to be cell centered and in focus. Cells are visualised using an inverted Nikon TE2000 microscope with a Nikon 60x/1.40 Plan Apo objective, a 430 nm LED light source (Cairn Research, Faversham, Kent UK) and a long-pass 455 nm dichroic mirror (DM455, Omega, Brattleboro, VT, USA). The reflected light is passed through a beam-splitter QuadView (Photometrics, Tucson, AZ, USA) to split the light into four channels before it reaches the camera.

The beam splitter has three long-pass dichroic mirrors: 514 nm, 552 nm, and 605 nm (Di02 R514, FF552 Di02, FF605 Di02, Semrock, Rochester, NY, USA), four bandpass filters: 433/24 nm 530/11 nm, 572/15 nm (centre/bandpass) (FF01-433/24, FF01-530/11, FF01-572/15 Semrock, Rochester, New York, USA), and a long-pass 700 nm filter (700LP, Omega, Brattleboro, VT, USA), for acquisition of cyan, yellow, orange and red light respectively. For the purposes of this paper, we only use two of these channels to acquire cyan and yellow light.

Recordings were made using an ORCA-Flash 4.0 camera (Hamamatsu Photonics, Welwyn Garden City, UK). The computer is equipped with an Intel(R) Core (TM) i7-4770 central processing unit (CPU), NVIDIA GeForce GT 630 graphical processing unit (GPU) and 16 GB of DDR3 random access memory (RAM). For full specifications see Supplementary A. The software for the recordings was Micro-Manager 1.4 (open source, Vale Lab, University of California, San Francisco, CA, USA), integrated into the Icy imaging suite (open source, http://icy.bioimageanalysis.org).

## 3. Results

### 3.1. Improvements Through New Real-Time FRET Software: “MultiFRET”

**Figure 1:**
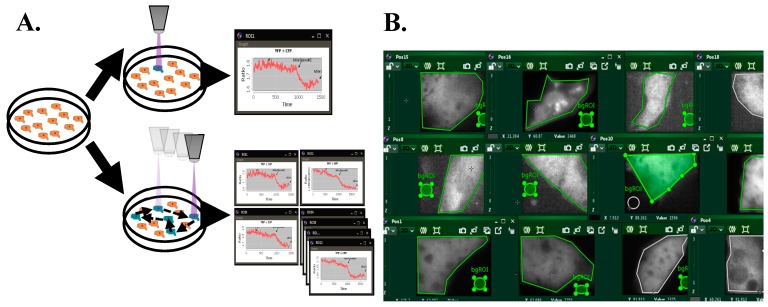
(**A**) Old method of single cell analysis (top) vs. new software (bottom). Using the new software, we can use a motorised stage to capture and analyse several cells simultaneously in real-time. At each time-interval of user-set length, the stage will cycle through all designated cells, capturing and analysing the respective data. This process is repeated after every interval. (**B**) Fluorescence intensity image set at one time-point showing the yellow channel of HiPCM-CM cells illustrating the multiple regions of interest that are measured simultaneously.

Our previous experimental setup was only able to record one field of view, containing, routinely, only one cell, resulting in a low throughput of one cell per hour (if an average experiment lasts an hour) or less (if the experimental protocol is longer). As a routine FRET experiment requires time-lapse acquisition of one frame every few seconds, we wished to increase the throughput by rapidly cycling between several preselected cells in the same dish. We have developed a new open-source Java plugin that increases the speed and ease of real-time FRET data acquisition in live cells. The MultiFRET plugin allows for a real-time high-throughput analysis as it enables live calculations from data acquired by moving the mechanical stage between cells and acquiring an image from each of them at set intervals (Figure 1).

The live analysis of FRET ratios in multiple cells allows users to add stimulants at a convenient point within the experiment’s cycle, avoiding potential artifacts. MultiFRET runs in Icy, an alternative to ImageJ with integrated Micro-Manager that aims to be both user-friendly and flexible enough to be used in any experiment requiring live acquisition and analysis of images. Additionally, processes run by the software are run in separate threads of execution, allowing e.g., fine adjustments to regions of interest at the beginning of an experiment as well as the simultaneous use of any other plugins. Both the raw image data and the analysed ROI data are saved, allowing reproduction of ratio-metric calculations offline if needed. Using this software, we have increased our experimental cell throughput from 1 to 25, and in our experiments this number has only been limited by the power of the computer running it and the speed and responsiveness of the motorised stage. As the software draws and updates a large number of images simultaneously, an appropriately powered GPU is required to render everything smoothly. At a maximum of 25 cells, we have not encountered any issues caused by lack of CPU or RAM. It can be expected that, as the number of cells increases beyond this point, the time taken by the stage to cycle through all the cells will become a limiting factor. While we could cycle through 25 cells, located within a 13 mm diameter round surface, well within 10 s, it may become a necessity to sacrifice time resolution for a higher number of cells to be recorded.

In addition, the MultiFRET plugin allows a user-friendly set-up of user-defined image correcting calculations, which can be put as a simple mathematical notation in the settings of the acquisition. Moreover, the plugin can export captured data into a user-designed Excel template for automated analysis of data.

### 3.2. FRET Calculations

Upon acquisition of each frame, the mean intensity of signal ($\bar{F})$ in each ROI is calculated following formula (1), where *n* is the number of pixels in ROI and *x* is the intensity of a single pixel *i*. These fluorescence signals are then used to calculate the FRET ratio in formula (2) by first subtracting background signal and then dividing the background-corrected donor signal by the background-corrected acceptor signal.

(1) $$\bar{F}=\frac{1}{n}\left(\overset{n}{\underset{i=1}{\sum }}x_{i}\right)$$

(2) $$\;\mathrm{FRET}=\;\frac{{\bar{F}}_{\mathrm{donor}}-{\bar{F}}_{\mathrm{donor}\;\mathrm{background}}}{{\bar{F}}_{\mathrm{acceptor}}-{\bar{F}}_{\mathrm{acceptor}\;\mathrm{background}}}$$

When data acquisition is completed, the data is sent to an Excel file containing formulae for the calculation of FRET response per stimulant. We first calculate a baseline plateau (*P*_base_) by calculating the mean of the FRET ratios of the last 10 acquisitions before adding the first stimulant. Then, for each stimulant, we first calculate a plateau (*P*_stim_) by calculating the mean of the FRET ratios of the last 10 acquisitions before adding a new stimulant. These means are used to calculate a change (*C*) from the baseline caused by each stimulant (3), this shift is then normalised to the change of the final plateau from base (*S*_max_) caused by addition of a signal maximiser and a % FRET shift is calculated (4).

(3) $$C=\frac{P_{\mathrm{base}}-P_{\mathrm{stim}}}{P_{\mathrm{base}}}$$

(4) $$\%\;FRET\;shift=\frac{100C_{\mathrm{stim}}}{C_{\max}}$$

### 3.3. Software Validation

**Figure 2:**
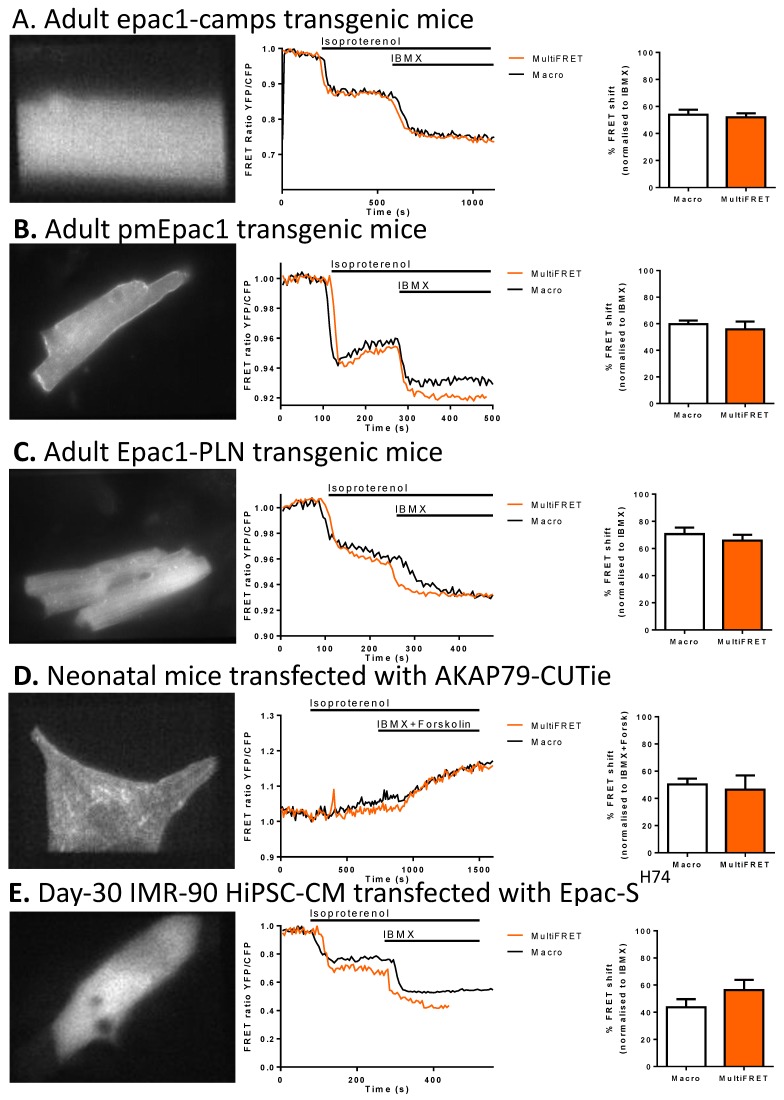
Software validation experiments showing the yellow channel with a representative cell (left panel), experimental trace and comparison of mean isoproterenol response between MultiFret and the old Macro. The means are normalised to the final plateau of IBMX or IBMX + Forskolin. (**A**) Adult mice transgenic for Epac1-camps, which causes a decrease in FRET efficiency upon binding cAMP (NS, n = 10). (**B**) Adult mice transgenic for pmEpac1, which causes a decrease in FRET efficiency upon binding cAMP. (NS; n = 10 for the Macro and n = 9 for MultiFRET). (**C**) Adult mice transgenic for Epac1-PLN, which causes a decrease in FRET efficiency upon binding cAMP (NS; n = 7). (**D**) Neonatal mouse CM transfected with the AKAP79-targeted CUTie sensor. This sensor brings fluorophores closer to each other upon binding of cAMP, showing an increase in the YFP/CFP ratio. In this experiment, IBMX (100 µM) and Forskolin (50 µM) were added together and data was normalised against the resulting plateau (NS; n = 6). (**E**) Day-30 IMR-90 hiPSC-CM transfected with Epac-S^H74^, which shows a decrease in FRET upon binding cAMP (NS; n = 20 for the Macro, and n = 19 for MultiFRET). For (**A**–**D**), data were acquired with the MultiFRET plugin and re-analysed with the ‘offline’ macro according to [13]. E macro data were obtained using the “online” macro and analysed using the “offline” macro according to [13], whereas MultiFRET data were acquired and analysed using MultiFRET with a different set of samples from the macro data. Data are shown as mean % FRET shift ± SEM. Welch’s test for unequal variances was used for statistics. Scale bars are 10 µm.

We validated our software by measuring FRET in various transgenic cardiomyocyte models expressing cytosolic and targeted cAMP sensors with both the new MultiFRET plugin and the old lower-throughput macro that has been used for FRET experiments in many previous studies. For the MultiFRET protocol, see Supplementary B. For our mouse experiments, we acquired the data using MultiFRET and then re-analysed the image-stacks using the Macro software’s “Offline” analysis. Comparing the software in adult epac1-camps transgenic mice stimulated with 30 nM β-adrenoceptor agonist isoproterenol and normalising against IBMX, we found no significant difference (Welch’s test for unequal variances) between the macro and MultiFRET (Figure 2A). Likewise, we could validate the new software using two targeted versions of this biosensor expressed in cardiomyocytes of transgenic mice. Both membrane targeted pmEpac1 and sarcoplasmic reticulum targeted Epac1-PLN biosensors showed similar responses to isoproterenol and IBMX measured with old and new software (Figure 2B,C). This is similar to experiments in neonatal AKAP79-CUTie mouse CMs, where, following the same experimental protocol, no significant difference was seen between the macro and MultiFRET (Figure 2D). For our hiPSC-CM experiments, we followed the same protocol with a difference in that the macro and MultiFRET data sets were acquired from separate samples. The macro dataset was acquired using the “Online” macro and analysed with the “Offline” macro. The MultiFRET data was acquired and analysed using MultiFRET. We again found no significant difference in FRET response between the two methods (Figure 2E). The minor differences in the representative traces are a result of the older macro cropping to a part of the image, possibly not showing the full cell. This fault in the old macro results in small differences in the areas analysed. Moreover, the ROI is selected separately between MultiFRET and the old macro, subjecting this to human error where the areas selected for analysis may be several pixels off.

## 4. Discussion

We have created the MultiFRET software with a modern and intuitive user interface (UI) that boasts flexibility, making it compatible with many experimental designs and system set-ups without the need to alter the software’s code. The plugin takes images acquired through Micro-Manager and immediately analyses them, showing a graph with ratio-metric analysis including background corrections in real time. This can be further combined with a mechanical X-Y-Z stage controlled through Micro-Manager yielding a graph for each position, allowing analysis of at least 25 cells per sample. Once the experiment is concluded, the acquired image data is stored to disk and the calculated ratio data is automatically output to an Excel file, which the user may set-up with a template, allowing immediate statistical analysis of their data. The high-throughput FRET increases data output per sample to 25-fold or more, decreasing costs and time spent on FRET experiments, while simultaneously allowing for comparison of cells within one sample. We experimentally validated our software by comparing it to the macro that has been used in previous studies for real-time ratio-metric FRET measurements, finding no significant differences. We also found that our data is in line with previously published data showing roughly a 40–60% FRET response of ISO stimulated AMVM transgenic for Epac1-camps normalised to IBMX [22]. While our validation experiments measure only two plateaus, up to just over 1500 s of recording, the length of the experiments does not affect the ability of the new or old software to measure and analyse individual data points.

Older software implementations often lack live analysis or ability to measure FRET in multiple cells [23]. We see this in the methods presented by S. Börner et al. [23] and J. U. Sprenger et al. [13], from which our current methods have sprung forth, where the experimental procedure is to measure FRET in a single cell but re-analyse this data subsequently using an “offline” analysis tool. This is far from optimal in terms of the experimenter’s time, and the design approach for MultiFRET was to minimise such inefficiencies as much as possible.

Another popular tool is Metafluor, this proprietary software allows for acquisition of multiple cells but only allows ratios to be measured in one region, limiting the number of cells per experiment and requiring a lower magnification to increase these numbers. Inconveniently, with Metafluor, there is also a separation between acquisition and final analysis by use of an “offline” tool.

There are more recently published methods such as laser scanning cytometer FRET and a technique based on total internal reflection fluorescence microscopy, which are capable of high-throughput FRET [7]. However, our software does not require specialized equipment and can be implemented on any microscope frame with a motorized x-y stage, where the frame and stage can be controlled through Micro-Manager. This allows for a cheaper solution as well as allowing the system to remain flexible, allowing the same light microscopy setup to be used with MultiFRET and other applications.

One major limitation as previously mentioned is the balance between time resolution and number of cells, as the mechanical stage needs to physically move to the next cell. At a high number of cells, this may affect experiments studying responses with faster dynamics than the ones demonstrated here. The hardware limitations mentioned can be mitigated to some degree through the use of a lower resolution of captured images and an upgrade of the GPU. Furthermore, a specific microscopy setup is needed to make full use of the software’s functions. However, even without the use of a mechanised stage and focus drive, the software still provides ease of use and automated analyses for any measurements performed on a single field of view. A key piece of equipment that the software will currently not run without is a beam-splitter, as such the software will not run on spinning disk microscopy setups. While our new MultiFRET software is flexible and easy to use, it currently only implements background correction.

In future, MultiFRET will be updated allowing the researcher to select the appropriate settings suitable for their machine and experimental design through implementation of a system to convert typical mathematical notation into real-time corrections utilized by the software. This will allow users to add corrections to the real-time analysis such as flat-fielding, bleed-through and any other user-defined corrections that could be required for a unique experimental set-up [24]. The plugin is freely available on request.

## Figures and Tables

**Table 1:** Forster Resonance Energy Transfer (FRET) sensors used in this study.

Sensor	Binding Domain	EC_50_	Reference
Epac1-camps	Epac1	2.4 µM	V.O. Nikolaev et al., J. Biol. Chem. 2004 [17]
pmEpac1	Epac1	9.1 µM	R.K. Perera et al., Circ. Res. 2015 [18]
Epac1-PLN	Epac1	5.3 µM	J.U. Sprenger et al., Nat. Commun. 2015 [19]
Epac-S^H74^	Epac1	11–14 µM	J. Klarenbeek et al., PLoS ONE 2011 [20]
AKAP79-CUTie	Prkar2β	7.4 µM	N. Surdo et al., Nature Commun 2017 [21]

## Supplementary Materials

The following are available online at https://www.mdpi.com/2073-4409/8/12/1541/s1, Supplementary file A. Specification of the computed used in this study. Supplementary file B. User manual for using MultiFRET plugin.
